# Using item response theory to enrich and expand the PROMIS^®^ pediatric self report banks

**DOI:** 10.1186/s12955-014-0160-x

**Published:** 2014-10-25

**Authors:** Hally Quinn, David Thissen, Yang Liu, Brooke Magnus, Jin-Shei Lai, Dagmar Amtmann, James W Varni, Heather E Gross, Darren A DeWalt

**Affiliations:** Department of Psychology, University of North Carolina at Chapel Hill, 358 Davie Hall, CB #3270, Chapel Hill, NC 27599 USA; Department of Medical Social Sciences, Northwestern University Feinberg School of Medicine, Chicago, IL USA; Department of Rehabilitation Medicine, University of Washington, Seattle, WA USA; Department of Pediatrics, College of Medicine, Department of Landscape Architecture and Urban Planning, College of Architecture, Texas A&M University, College Station, TX USA; Cecil G. Sheps Center for Health Services Research, University of North Carolina at Chapel Hill, Chapel Hill, NC USA; Division of General Medicine and Clinical Epidemiology, Cecil G. Sheps Center for Health Services Research, University of North Carolina at Chapel Hill, Chapel Hill, NC USA

**Keywords:** PROMIS, Pediatrics, Self-report, Patient reported outcomes, Item response theory

## Abstract

**Background:**

The primary objective was to enhance the content coverage of some of the pediatric self-report item banks for ages 8–17 years from the National Institutes of Health (NIH) Patient Reported Outcomes Measurement Information System (PROMIS^®^), and extend the range of precise measurement to higher levels of physical functioning.

**Methods:**

Data from 1,419 pediatric patients with cancer, chronic kidney disease, obesity, rehabilitation needs, rheumatic disease, and sickle cell disease were combined with item responses from the original standardization sample of 3,048 children to calibrate new items for the pediatric PROMIS Anger, Anxiety, Depressive Symptoms, Pain Interference, Fatigue, and physical functioning Upper Extremity and Mobility scales. Simultaneous or concurrent calibration using the graded item response theory model placed all of the items on the same scale.

**Results:**

Twenty-two of 28 potential new items were added across the seven scales. A recommended short form was proposed for the Anger scale, and the recommended short forms for the Anxiety and Depressive Symptoms scales were revised. Unfortunately, we were not particularly successful at extending the range of measurement for the physical functioning banks.

**Conclusions:**

The present study expanded PROMIS pediatric item banks to add new content and to increase the range of measurement. Using item response theory, the banks were revised and expanded without changing the underlying scale of measurement. For Anger, Anxiety, and Depressive Symptoms, we successfully added new content that may render those banks more robust and flexible.

**Electronic supplementary material:**

The online version of this article (doi:10.1186/s12955-014-0160-x) contains supplementary material, which is available to authorized users.

## Background

The Patient Reported Outcomes Measurement Information System (PROMIS^®^) was created to advance the assessment of patient-reported outcomes (PRO) in patients with chronic diseases. A primary objective was to develop item banks, which would support short forms and computerized adaptive tests (CATs) that could be administered to patients with a variety of chronic health conditions [1]. The PROMIS Pediatric Working Group created self-report item banks for ages 8–17 years across five general health domains (emotional health, pain, fatigue, physical function, and social health), consistent with the larger PROMIS network [2]. The PROMIS pediatric measures were developed using qualitative and quantitative methods. The procedures involved the use of focus groups, expert item review, cognitive interviewing, item administration to a large population of children and adolescents, and item response theory (IRT) analyses to create banks of items specific to selected domains [3-5].

Although the PROMIS pediatric measures were successfully created and are currently being used in research, there were several areas for improvement. Among the emotional distress scales, the final Anger item bank included only six items, which limited its precision. The Anxiety and Depressive Symptoms item banks were 15 and 14 items, respectively [6,7]; and the Pain Interference item bank included only 13 items [8]. Although the original banks had acceptable precision, we sought to extend the range of the measured latent trait covered by items in the bank to minimize floor and ceiling effects. Although the two physical functioning item banks had larger numbers of items, 29 for Upper Extremity and 23 for Mobility, the information provided by those items was concentrated in the lower range of physical functioning [9]. While that is appropriate for many uses of the scales in health outcomes research, we wanted to add items that might extend the range of precise measurement to higher levels of physical functioning.

The PROMIS Pediatric Anger scale comprised the entire bank of 6 items. After expanding the Anger pool, a useful short form could be created. The recommended short forms for the Anxiety and Depressive Symptoms banks also required revision, both to incorporate new items that may prove more useful than existing items, and to remove items that were not optimal.

With these goals in mind, 28 potential new items were developed and administered along with the existing item banks to test whether they could be added to existing item banks and enhance content and range of measurement.

## Methods

### The chronic illness sample

Data collection across the samples took place during a 1-year period from 2009 to 2010. Participants were recruited from hospital-based general pediatric clinics, subspecialty clinics, and hospital inpatient units. Participants were identified through a review of medical charts, clinic appointment rosters or while in the clinic waiting rooms according to protocols approved by the institutional review boards (IRBs) at each of the participating institutions. The data in the present study include previously published or submitted data on individual disease groups [10-12]. However, analysis of the responses to the potential new items has not been described.

To be eligible to participate in the study, all participants were required to meet the following inclusion criteria: able to speak and read English; able to see and interact with a computer screen, keyboard, and mouse; and were between the ages of 8 and 17 years. The exclusion criteria were children having any concurrent medical or psychiatric condition that might preclude participation in this study or cognitive or other impairment (e.g., visual) that would interfere with completing a self-administered computer based questionnaire. Parents signed an informed consent document and children signed an informed assent document that outlined the following: purpose of the study, participation requirements, potential benefits and risks of participation, and the measures implemented to protect participant privacy. Both the informed assent and the informed consent were administered in English, so parents were also required to read and speak English. Each participant received a $10 gift card in return for his or her time and effort.

There were six disease-specific subsamples:

- *Cancer sample.* Pediatric patients were recruited from the Children’s National Medical Center in Washington, DC; Nebraska Medical Center in Omaha, Nebraska; Children’s Hospital Los Angeles in California; Palmetto Health Children’s Hospital in Columbia, South Carolina and Emory University in Atlanta, Georgia. In addition to the general inclusion and exclusion criteria cited above, pediatric patients were considered eligible for study enrollment if they were currently receiving curative cancer treatment (defined as disease-directed therapy within the past 45 days) or had completed cancer treatment and were disease-free and in follow-up care (survivorship group). An additional exclusion criterion included patients who were receiving end-of-life care (defined as supportive treatment following a decision against resuscitation or favoring terminal care with possible hospice involvement). A total of 200 cancer patients participated.
- *Chronic kidney disease sample.* Pediatric patients were recruited through the Midwest Pediatric Nephrology Consortium from 16 participating member institutions. In addition to the general inclusion and exclusion criteria cited above, pediatric patients who were considered eligible for study enrollment had existing chronic kidney disease, defined as dialysis or kidney transplant dependence, estimated glomerular filtration rate (eGFR) <90 ml/min/1.73 m^2^ or nephrotic syndrome [13]. An eGFR ≤15 was chosen to represent kidney failure. In total, 384 children with chronic kidney disease were participants in this study.
- *Obesity sample.* Pediatric patients were recruited from five participating sites including an academic obesity clinic, three private pediatric practices and a federally qualified health center in North Carolina. In addition to the general inclusion and exclusion criteria cited above, pediatric patients who were considered eligible for study enrollment had an age adjusted body mass index (BMI) ≥85th percentile. A total of 136 obese patients participated.
- *Rehabilitation sample.* Patients with rehabilitation needs were recruited from the participant pool of other studies at the University of Washington, clinics at Seattle Children’s Hospital, and study advertisements. There were 102 patients in the rehabilitation sample, including 22 who had spina bifida (21.6%), 20 with cerebral palsy (19.6%), 17 with neuromuscular disease (16.7%), 12 who had limb differences (11.8%), 6 who had experienced traumatic brain injuries (5.9%), and 25 with other diagnoses (24.5%).
- *Rheumatic disease sample.* Patients with rheumatic disease were recruited from rheumatology clinics at four academic medical centers in California, North Carolina, Ohio, and Washington State. In addition to the general inclusion and exclusion criteria cited above, patients needed to have a physician-confirmed diagnosis of juvenile idiopathic arthritis (JIA), childhood systemic lupus erythematosus (cSLE), juvenile dermatomyositis (JDM), or overlapping conditions. A total of 362 patients participated, 269 (74.3%) with JIA, 42 (11.6%) with cSLE, 23 (6.3%) with JDM, and 28 (7.7%) with overlapping conditions.
- *Sickle cell disease sample.* Pediatric patients were recruited from two large East Coast sickle cell disease programs (Emory University, Duke University). In addition to the general inclusion and exclusion criteria cited above, pediatric patients were considered eligible for study enrollment if they received a physician diagnosis of sickle cell disease. Participants were recruited at clinic visits for routine care, hydroxyurea monitoring, or for chronic transfusions. At the time of the study, 19.1% of the participants were receiving chronic transfusions and 45.5% were taking hydroxyurea. A total of 235 patients participated.

### The original standardization sample

The original standardization sample for the PROMIS Pediatric scales included 3,048 children; a detailed description of the sample has been provided [3]. The general eligibility criteria were the same as for the chronic illness sample. Parent report was used to determine whether or not the child had any limitations (e.g., physical or cognitive) that would make it too difficult to complete a computer-administered survey.

Participants were recruited in public school settings and hospital-based outpatient general pediatrics and subspecialty clinics. According to protocols approved by the institutional review boards (IRBs) of The Children’s Hospital at Scott and White (S&W) in Texas, the University of North Carolina (UNC), and Duke University pediatrics clinics, potential clinic participants were identified through a variety of methods such as review of pediatric clinic appointment rosters or while in the clinic waiting rooms. The children recruited in the UNC, Duke, and S&W general pediatric clinics had typical health issues for which children have physician office visits (e.g., well child visits, acute illnesses, as well as some chronic illnesses). The specialty clinics included pulmonology, allergy, gastroenterology, rehabilitation, rheumatology, nephrology, obesity, and endocrinology and primarily saw children with more serious chronic illnesses.

School-based participants were recruited through the Chapel Hill-Carrboro (NC) Public School System, including elementary after school programs as well as required middle and high school health classes. An informational packet was mailed to all of the parents with children enrolled in the health classes to inform them about the study. This packet contained general information about the study, the informed consent documents, and parental forms (sociodemographic form) to complete and return to the school.

Parents signed an informed consent document and children signed an informed assent document that outlined the following: purpose of the study, participation requirements, potential benefits and risks of participation and measures implemented to protect participant privacy. The institutional review boards at each institution approved the study protocols. Data were collected between January 2007 and May 2008.

#### Pediatric self-report item banks and potential additional items

Items from the PROMIS pediatric Anger, Anxiety, Depressive Symptoms, Pain Interference, Fatigue, and physical functioning Upper Extremity and Mobility scales are considered in this study. Participants in the Chronic Illness sample were administered a combination of short forms and/or complete item banks, with different combinations for the condition-specific subsamples. The potential new items were embedded among the existing items in the computerized administration.

For each scale, higher scores indicate more of the construct being measured. For example, higher scores on the Emotional Distress Scales indicate more (worse) emotional distress; higher scores on the Physical Functioning Scales indicate higher (better) levels of physical functioning. All items had a 7-day recall period and used standardized 5-point response options (e.g., *never*, *almost never*, *sometimes*, *often*, *almost always*; or, *with no trouble*, *with a little trouble*, *with some trouble*, *with a lot of trouble*, *not able to do* for physical functioning scales).

The candidate new items were developed based on known limitations of the original item banks (e.g., the physical function banks had substantial ceiling effects and we desired more items of greater difficulty; the numbers of existing and new items are shown in Table 1). After drafting several new items to cover a broader range of the trait or to fill potential content gaps, items were subjected to the same cognitive interviewing protocol as the original items [5]. Each item was reviewed in detail by a minimum of 5 children between the ages of 8 and 17. Items that were difficult to understand or interpreted differently than intended, were discarded or reworded and subjected to additional cognitive interviews.

**Table 1:** **The number of items in each of seven PROMIS pediatric item pools, the number of new items considered for addition, and the disposition of the new items**

	**Number of items**				
**Domain**	**Original item pool**	**New items considered**	**New items after dimensionality analysis**	**New items after DIF analysis and calibration**	**Enlarged item pool**
Anger	6	5	5	4	9^†^
Anxiety	15	5	3	2	15^††^
Depressive symptoms	14	1	1	1	14^†††^
Pain interference	13	7	7	7	20
Fatigue	23	2	2	2	25
Physical functioning: upper extremity	29	5	5*	5	34
Physical functioning: Mobility	26	3	3	1	27

^†^One item was removed from the original Anger item pool due to conflicting copyright claims.

^††^Two items were removed from the original Anxiety item pool due to conflicting copyright claims.

^†††^One item was removed from the original Depressive Symptoms item pool due to conflicting copyright claims.

*Two new items exhibited LD, but were retained as “enemy items” to expand the range of the scale for those with higher physical functioning.

### Statistical analysis

#### Preliminary checks on the data

As preliminary checks on the validity of the data, traditional test theory descriptive statistics were computed to verify that there were no empty (zero frequency) response categories for any item, within any of the groups of participants. Marginal frequencies of item responses and correlations of item scores with the total summed score were also computed and examined.

#### Checking dimensionality

The graded response IRT model [14,15] that is used here for item analysis and scoring is based on the assumption that responses to the items indicate individual differences on a single underlying, or latent, variable for each scale. To select items measuring a single variable without contamination by other constructs, the data analysis used several approaches to check for local dependence (LD) or other evidence of multidimensionality in the data. The first approach used the approximately standardized *LD X*^2^ statistics [16] reported by the computer software IRTPRO [17] as diagnostic statistics for unidimensional IRT models fitted to all of the existing and potential new items for each scale. If values were over 5 then item content of relevant pairs was examined to consider whether the items were sufficiently similar to yield LD. If items were judged to represent similar content, then confirmatory item factor analysis (CFA) with a bifactor model was fitted with second-tier factors representing the LD pairs. Such CFA models are also called multidimensional IRT (MIRT) models. The models were fitted with the IRTPRO software [17], with non-zero MIRT slopes or factor loadings for the LD items, and fixed zeros for all other items, for all factors but the first general factor. The ratios of the fitted second-tier slopes (or loadings) to their standard errors were used as large-sample *z-*statistics to test the significance of the LD.

For some scales with very skewed distributions of observed item responses, the *LD X*^2^ statistics fail to suggest a clear pattern of LD or multidimensionality. Exploratory item factor analysis (EFA) was used instead to give LD, or additional dimensions, an opportunity to become visible. For the scales with larger numbers of items, EFAs included up to three factors; patterns of loadings across those factors could suggest bifactor models of even higher dimensionality, which were then fitted. If the fitted bifactor models included second-tier slopes or loadings that did not differ significantly from zero, the models were refined by fixing those values at zero and re-fit. Again, the ratios of the fitted second-tier slopes (or loadings) to their standard errors were used as Wald statistics to test the significance of the LD or narrower second-tier factors.

For item sets that exhibited multidimensionality, the value of explained common variance (ECV) [18] for the general factor was computed from each final bifactor model. The ECV indicates the proximity of the data to unidimensionality (an ECV value of 1).

The final judgment whether an item, or cluster of items, was to be set aside or retained was made following discussion among the authors. Statistical evidence of multidimensionality and measures of its effect size, the apparent similarity of the content of the items in the pair or cluster, and whether the items were “new” or already on the scale, were considered simultaneously. Measures of effect size included the MIRT slope values, the factor loading estimates for the items on the second tier factors, and the product of the factor loadings, which is the contribution to the between item correlation due to LD. New items were set aside with less evidence of LD than was required for items already on the scales.

#### Calibration—IRT parameter estimation for the new items

After setting aside items judged to exhibit LD, IRT parameters for the potential new items were estimated using concurrent calibration with the existing items on each scale. To place all parameters on the original scale, the subset of the original standardization sample from UNC served as the reference population (calibration scale: mean = 0, SD =1; reporting scale: mean = 50, SD = 10). This set the location and intervals for the original scores. The rest of the data (the Texas portion of the original calibration sample and the entire chronic illness sample) were combined into a single additional group with an estimated mean and standard deviation.

In parallel with these item calibrations, IRT-based analyses of differential item functioning (DIF) checked whether the item parameter estimates for the potential new items differed significantly by sex or age (8–12 years vs. 13 = 17 years), with the original items for each scale serving as the “anchor” [19]. DIF analysis checked for another kind of evidence of a lack of unidimensionality of item responses. Items that exhibited significant DIF were considered carefully and possibly set aside, instead of being added to the scales.

#### Creation and revision of short forms

After the addition of new items to the Anger bank, there were enough items for a recommended short form to be selected, following the same procedures used in the construction of the original banks: We used the IRT estimates of information at each level of the underlying latent variable as statistical evidence about the relative usefulness of each item, as well as judgment to select items spanning the range of content, in order to select a useful set of eight items with information covering a wide range of the construct. We also revised the recommended short forms for the Anxiety and Depressive Symptoms banks.

## Results

### The new items

Of the 28 potential new items considered, 22 were added to their respective item banks. Table 1 provides a summary of the numbers of items originally included in each of the seven item banks considered here, and the distribution of outcomes for the potential new items for each scale. The remainder of this section describes the results for each scale separately, referring to the numerical results in Tables 2, 3, 4.

**Table 2:** **Item parameters and values for the** ***SS X*** ^**2**^ **fit index and LR DIF statistics for the potential new items for the anger, anxiety, and depressive symptoms scales**

									**DIF between**			
	**Item parameters**					***S*** **-** ***X*** ^**2**^ **fit index**			**Boys and Girls**		**Ages**	
Item Stem	*a*	*b*_1_	*b*_2_	*b*_3_	*b*_4_	*X* ^2^	*d.f.*	*p*	*X* ^2^	*p*	*X* ^2^	*p*
*Added to the Anger item bank:*												
I was angry when things didn’t go my way	1.41	−0.8	0.33	1.74	2.60	62	70	0.748	2.6	0.755	5.8	0.328
I wanted to be alone because I was so angry	1.92	−0.51	0.39	1.56	2.64	62	61	0.428	1.3	0.932	3.5	0.621
I was so mad I did not want to talk to people	2.1	−0.25	0.51	1.86	3.01	54	53	0.448	5.3	0.382	2.5	0.772
I had a bad temper	1.69	−0.44	0.60	1.71	2.64	62	67	0.640	9.9	0.079	2.9	0.710
*Not added to the Anger item bank (DIF):*												
I could not control my anger	1.69	0.11	0.96	1.86	2.36	785	59	0.050	**16.4**	**0.006**	9.1	0.104
*Added to the Anxiety item bank:*												
I felt too nervous to be with a group of kids my age.	1.34	0.52	1.42	2.81	3.48	77	75	0.401	2.4	0.794	2.0	0.856
I worried that something might happen to my parents or guardians.	1.29	−0.23	0.45	1.73	2.28	121	92	0.024	5.9	0.314	2.1	0.840
*Not added to the Anxiety item bank (DIF):*												
I was too worried to sleep alone.	1.34	0.93	1.79	2.65	3.32	83	60	0.026	3.8	0.576	**25.1**	**0.001**
*Not added to the Anxiety item bank (LD):*												
I had trouble falling asleep because I was worried about something.	1.65	−0.13	0.72	2.14	2.91	78	74	0.343	7.6	0.176	3.0	0.707
I was so nervous I felt sick.	1.40	0.59	1.29	2.80	3.86	80	72	0.2332	0.6	0.989	4.1	0.537
*Added to the Depressive Symptoms item bank:*												
I felt sad for no reason.	1.65	0.41	1.19	2.36	3.31	82	70	0.160	7.2	0.124^†^	4.9	0.427

Notes: The scale for the item parameters is set such that the distribution of emotional distress in the reference population (represented by the NC portion of the sample) is standardized, mean 0 variance 1, as is conventional for reporting IRT parameters. *d.f.* =5 for all DIF *X* ^2^ statistics except that marked with ^†^, for which *d.f.* = 4. Values in bold indicate test statistics and p values that are significant at *p* < .05 after adjustment for multiple comparisons.

**Table 3:** **Item parameters and values for the** ***SS X*** ^**2**^ **fit index and LR DIF statistics for the potential new items for the pain interference and fatigue scales**

									**DIF between**			
	**Item parameters**					***S*** **-** ***X*** ^**2**^ **fit index**			**Boys and Girls**		**Ages**	
Item Stem	*a*	*b*_1_	*b*_2_	*b*_3_	*b*_4_	*X* ^2^	*d.f.*	*p*	*X* ^2^	*p*	*X* ^2^	*p*
*Added to the Pain Interference item bank:*												
It was hard for me to be away from home because I had pain.	2.14	0.36	0.8	1.63	2.18	118	88	0.020	12.6	0.027	10.2	0.070
It was hard to have fun with friends because I was in pain.	2.79	−0.06	0.48	1.3	2.12	107	81	0.028	8	0.154	11.7	0.040
I needed help walking when I was in pain.	2.18	0.4	1.04	1.87	2.35	84	80	0.358	0.9	0.970	3.4	0.640
I walked carefully when I was in pain.	1.92	−0.41	0.1	1	1.65	171	107	0.001	8.1	0.149	4.1	0.531
I had so much pain I had to stop what I was doing.	2.6	−0.07	0.53	1.61	2.27	82	83	0.524	4.6	0.472	10.7	0.058
My pain was so bad that I needed to take medicine to treat it.	1.9	−0.21	0.28	1.08	1.74	112	101	0.211	5.5	0.356	9.9	0.077
It was hard to do things with my family because I had pain.	2.79	0.08	0.61	1.77	2.33	75	72	0.388	3.4	0.647	8.3	0.138
*Added to the Fatigue item bank:*												
Being tired made it hard for me to remember things.	1.56	−0.2	0.67	2.26	3.05	105	94	0.203	3	0.694	1.9	0.861
I felt tired even when I had not done anything.	1.4	−0.46	0.46	1.87	3	105	102	0.396	4.6	0.469	6.2	0.284

Notes: The scale for the item parameters is set such that the distribution of pain interference and fatigue in the reference population (represented by the NC portion of the sample) is standardized, mean 0 variance 1, as is conventional for reporting IRT parameters. *d.f.* = 5 for all DIF *X* ^2^ statistics.

**Table 4:** **Item parameters and values for the** ***SS X*** ^**2**^ **fit index and LR DIF statistics for the potential new items for the physical functioning: upper extremity and mobility scales**

									**DIF between**			
	**Item parameters**					***S*** **-** ***X*** ^**2**^ **fit index**			**Boys and Girls**		**Ages**	
Item Stem	*a*	*b*_1_	*b*_2_	*b*_3_	*b*_4_	*X* ^2^	*d.f.*	*p*	*X* ^2^	*p*	*X* ^2^	*p*
*Added to the Upper Extremity item bank:*												
I could pick up coins from a table.	2.94		−3.23	−2.66	−1.87	10	8	0.247	3.8	0.291^††^	2.9	0.406^††^
I could wash my hair by myself.	2.14		−2.84	−2.37	−1.61	18	22	0.731	1.3	0.861^†^	5.4	0.253^†^
I could put beads on a string.*	1.95	−3.32	−3.04	−2.41	−1.56	30	23	0.151	6.3	0.283	5.9	0.212^†^
I could comb my hair by myself.	2.43	−3.25	−2.8	−2.59	−1.65	19	14	0.160	3.9	0.269^††^	5	0.286^†^
I could thread a needle.*	1.54	−2.14	−2.02	−1.43	−0.57	50	41	0.162	9.7	0.083	12.1	0.034
*Added to the Mobility item bank:*												
I could jump up and down.	2.93	−2.3	−2.06	−1.7	−1.16	73	43	0.003	4.1	0.539	2.5	0.774
*Not added to the Mobility item bank (DIF):*												
I could get in and out of a chair on my own.	3.46	−3.06	−2.79	−2.38	−1.78	32	16	0.010	3.3	0.657	**24.6**	**0.001**
*Not added to the Mobility item bank (LD and DIF):*												
I could run three miles without stopping.	1.51	−0.52	0.04	1.04	1.74	52	57	0.678	**14.8**	**0.011**	7.6	0.176

Notes: The scale for the item parameters is set such that the distribution of physical functioning in the reference population (represented by the NC portion of the sample) is standardized, mean 0 variance 1, as is conventional for reporting IRT parameters. *d.f.* =5 for all DIF *X* ^2^ statistics except those marked with ^†^, for which *d.f.* = 4, and those marked with ^††^, for which *d.f.* = 3. Values in bold indicate test statistics and p values that are significant at *p* < .05 after adjustment for multiple comparisons.

*Marked as “enemy items” with each other, and with “I could put toothpaste on my toothbrush by myself” from the original item pool.

#### Anger

Neither *LD X*^2^ statistics computed in the course of concurrent unidimensional calibration of the augmented Anger item pool, nor subsequent fitting with a bifactor model, suggested evidence of substantial local dependence or deviation from unidimensionality. No additional factor analyses were performed on this item set. DIF analysis revealed significant DIF between boys and girls for the new item “I could not control my anger,” so that item was set aside. The remaining four new items were added to the Anger item pool (Table 2).

#### Anxiety

The *LD X*^2^ statistics computed in the course of concurrent unidimensional calibration of the five new items with the existing 15-item Anxiety item pool suggested local dependence for six pairs of items, three of which involved the new items and three were pairs of items in the existing pool (*LD X*^2^ values range 6.1 - 19.9). Confirmatory factor analysis using a restricted MIRT model revealed that second tier bifactor loadings associated with five of those six locally dependent pairs were significantly greater than zero. Two of the new items were set aside due to redundancy (LD) with items in the existing pool: “I had trouble falling asleep because I was worried about something” with the existing “I worried when I went to bed at night”, and “I was so nervous I felt sick” with “I felt nervous” from the original scale. In addition, the new item “I was too worried to sleep alone” exhibited DIF between younger and older children, and was set aside for that reason. After these analyses, two items were added to the Anxiety item pool (Table 2).

#### Depressive symptoms

Diagnostic statistics suggested no evidence of local dependence involving the single new item (e.g., I felt sad for no reason), and DIF analysis revealed no significant DIF for that item. This item was added to the Depressive Symptoms item pool (Table 2).

#### Pain interference

*LD X*^2^ statistics computed in the course of concurrent unidimensional calibration of the seven new items with the existing 13-item Pain Interference item pool suggested local dependence among some of the existing items, with values from 6.7 to 13 and one pair that involved a new item. Confirmatory factor analysis using a bifactor MIRT model indicated that the LD, while marginally significant, was at a very low level; the value of ECV for the 20-item set was 0.87, suggesting a close approximation to unidimensionality. The contribution of the second tier factors to the inter-item correlations ranged from 0.05 to 0.12; this was considered negligible. After these analyses, all seven new items were added to the Pain Interference item pool (Table 3).

#### Fatigue

The *LD X*^2^ statistics computed in the course of concurrent unidimensional calibration of the two new items with the existing 23-item Fatigue item pool did not show any clear pattern, so exploratory item factor analysis was used to investigate potential multidimensionality. Confirmatory factor analysis using a bifactor MIRT model, based on suggestions from the 3-factor EFA, indicated some degree of multidimensionality, but at a very low level; the value of ECV for the 25-item set was 0.8, suggesting a sufficiently good approximation to unidimensionality. Both new items were added to the Fatigue item pool (Table 3).

#### Physical functioning: upper extremity

A sequence of analyses using *LD X*^2^ statistics computed in the course of concurrent unidimensional calibration of the five new items with the existing 29-item Upper Extremity item pool, exploratory item factor analysis, and fitting increasingly refined bifactor models led to the conclusion that two of the new items were involved in an LD triplet with one of the existing items. The triplet included the new items “I could thread a needle”, “I could put beads on a string”, and the old item “I could put toothpaste on my toothbrush by myself.” However, the goal in adding items to this scale was to expand the range of measurement of the scale toward higher levels of physical functioning, and those two new items do that to some extent, as indicated by their IRT information functions. Therefore, the decision was made to add those two items to the pool, with the annotation that any CAT or user-constructed forms include only one member of that locally dependent triplet to avoid the LD in scoring. (In the CAT literature this is sometimes called marking items as “enemies.”) The other three new items appeared to be unidimensional with the rest of the scale, and did not exhibit significant DIF with respect to respondent sex or age. As a result, all five new items were added to the scale, with the caveat about the two “enemy” items (Table 4).

#### Physical functioning: mobility

A similar sequence of analyses to that for the other physical function scale, using *LD X*^2^ statistics computed in the course of concurrent unidimensional calibration of the three new items with the existing 23-item Mobility item pool, exploratory item factor analysis, and fitting increasingly refined bifactor models, led to the conclusion that one of the new items was involved in an LD pair with one of the existing items. The pair was “I could run three miles without stopping” (new) with “I could run a mile” (existing). “I could run three miles without stopping” had been proposed to extend the range of the scale, with some LD expected. But it also turned out to be less informative than most existing items on the scale, and it exhibited DIF between boys and girls, so it was set aside. In addition, the item “I could get in and out of a chair on my own” exhibited DIF by age, and was also set aside. Consequently, the only item added to the Mobility item pool was “I could jump up and down” (Table 4).

### The anger short form, and revision of the anxiety and depressive symptoms short forms

After adding four new items to the original six-item Anger item bank, and removing one item due to similarities with an item from another scale, the Anger item bank has nine items, which is one more than the eight-item length for recommended short forms for most of the PROMIS pediatric scales. It is therefore useful to recommend an eight-item set to serve as a standard short form for the PROMIS Pediatric Anger scale. The single item from the bank that is not used on this short form is “I felt fed up”, which the IRT analysis indicates provides the least information among the anger items. The items for the recommended Anger short form are in the Additional file 1, along with a score conversion table based on IRT that converts summed scores into the corresponding scale scores using the standard PROMIS metric with a midpoint of 50 and standard deviation 10. The marginal reliability of the converted summed scores is 0.85.

The Additional file 1 also includes revised recommended short forms for the Anxiety and Depressive Symptoms scales, along with scoring tables based on IRT that convert summed scores into the corresponding scale scores. These revised short forms replace three items (two for Anxiety and one for Depressive Symptoms) that are removed because of similarities with other scales. These items were replaced with the next-most-informative items selected from among the combined new and original items that now comprise the two banks. In both cases, items from the original calibration were selected as replacements. The marginal reliability of the converted summed scores is 0.83 for the Anxiety scale and 0.85 for the Depressive Symptoms scale.

## Discussion

This study illustrates the use of IRT to maintain and expand item banks for health outcomes measures. We successfully added new items to the Anger, Anxiety, and Depressive Symptoms banks. By adding these new items, we have increased the potential precision of the Anger measure and added additional content to the Anxiety and Depressive Symptoms banks. Unfortunately, we were not particularly successful at extending the range of measurement for the physical functioning banks. While six items were added to the physical functioning banks, those items did not turn out to provide much more information at the higher levels of physical functioning than the pre-existing items had. The *b* parameters of the new items did not reach higher levels than those of existing items, meaning no more information was added there (see Table 4). It is not clear that higher-than-average levels of physical functioning can be measured with a unidimensional scale; persons who achieve higher-than-average levels of physical functioning may do so in a variety of ways (e.g., running long distances, participating in a variety of exercises, like biking, running, and hiking), rendering the reported data about their performance multidimensional. This is certainly a subject meriting further research; however, at this time, precision in the measurement of general physical functioning remains limited to the lower ranges of performance.

In this paper we also illustrate the flexibility of PROMIS measures by recommending new short forms for Anger, Anxiety, and Depressive Symptoms. For Anger, the new short form reflects the substantial expansion of the item bank. For Anxiety and Depressive Symptoms, the new short form reflects the removal of some items; we are able to replace them while still maintaining our original measurement properties.

Scales based on item response theory are dynamic instruments; alternate forms and computerized adaptive tests can be created from existing item banks, and those item banks can be revised and expanded without changing the underlying scale of measurement. Revisions that involve removal of some items or additions of others can still yield scores that are comparable with results obtained with earlier versions. This feature of IRT has been used for the past two decades in educational measurement, to provide trend data based on evolving tests of academic achievement; the PROMIS scales are among the first to bring this modern test theory to health outcomes measurement.

We have illustrated one way that IRT can be used to add items to a bank, using concurrent calibration of the (potential) new items with the original item response data that was used as the basis of scale construction. Intuitively, this procedure basically retroactively adds the new items to the original scale; it is as though they had been there in the first place. In this case, we left the original item parameters unchanged, because they are already in use, and there is little to be gained by replacing those parameters. However, we observe in passing that it was a choice to do that. The original items also had a set of new parameters that were obtained in this concurrent calibration with the new items. Those parameter estimates are not used for anything beyond this particular analysis, although eventually they could be used to check for item parameter drift over time.

There are other ways that IRT procedures can be used to add new items to existing scales. Von Davier and von Davier provide a theoretical integration of a number of methods that have been developed over the past twenty-five years, largely in the context of educational measurement [20]. Of these methods, concurrent calibration imposes the fewest arbitrary restrictions on maximum likelihood item parameter estimation, so it is preferable where feasible. However, the use of the original calibration data may present obstacles in some situations. In such instances, calibration of the new items using fixed item parameters for the original items, or the use of the Stocking-Lord procedure to combine separate calibrations, may also be useful [21]. IRT provides potential solutions for most measurement problems, which is one of the reasons the newly-developed PROMIS scales are so useful.

## Conclusions

We successfully expanded content in the PROMIS pediatric item banks using IRT. Although we did not substantially reduce ceiling and floor effects, we have made more diverse content available to researchers and further optimized short forms for Anger, Anxiety, and Depressive Symptoms. These methods demonstrate the usefulness of IRT for continually enhancing health measurement while maintaining a consistent underlying measurement system. Future researchers and clinicians using the PROMIS scales will benefit from having an expanded pool from which to select their items.

## Additional file

Additional file 1:
**Appendix.** The additional file includes the item stems and scoring tables for the revised recommended eight-item short forms for the PROMIS Pediatric Anxiety and Depressive Symptoms Scales, and the recommended eight-item short form for the PROMIS Pediatric Anger Scale. All items use a 7-day recall period (the preface is “In the past seven days”), and a 5-point response scale with the options *never* (0), *almost never* (1), *sometimes* (2), *often* (3) and *almost always* (4).

## Abbreviations

PROMIS^®^: Patient Reported Outcomes Measurement Information System
NIH: National Institutes of Health
