# Supplementary material for: Using item response theory to enrich and expand the PROMIS® pediatric self report banks
Source: Health Qual Life Outcomes. 2014 Oct 25;12:160. doi: 10.1186/s12955-014-0160-x (PMC4212129; doi:10.1186/s12955-014-0160-x)
Supplement: Additional file 1: — Appendix. The additional file includes the item stems and scoring tables for the revised recommended eight-item short forms for the PROMIS Pediatric Anxiety and Depressive Symptoms Scales, and the recommended eight-item short form for the PROMIS Pediatric Anger Scale. All items use a 7-day recall period (the preface is “In the past seven days”), and a 5-point response scale with the options never (0), almost never (1), sometimes (2), often (3) and almost always (4). [file 12955_2014_160_MOESM1_ESM.docx]

**Additional file 1**

**Appendix** Listed below are the item stems for the revised recommended eight-item short forms for the PROMIS Pediatric Anxiety and Depressive Symptoms Scales, and the recommended eight-item short form for the PROMIS Pediatric Anger Scale. All items use a 7-day recall period (the preface is “In the past seven days”), and a 5-point response scale with the options *never* (0), *almost never* (1), *sometimes* (2), *often* (3) and *almost always* (4).

Anxiety:

I felt scared.

I worried about what could happen to me.

I felt worried.

I felt like something awful might happen.

I worried when I went to bed at night.

I felt nervous.

I got scared really easy.^†^

I worried when I was at home.^†^

^†^These items replace “I thought about scary things” and “I was afraid that I would make mistakes” on the original recommended short form.

Depressive Symptoms:

I felt like I couldn’t do anything right.

I felt everything in my life went wrong.

I felt unhappy.

I felt lonely.

I felt sad.

I felt alone.

I could not stop feeling sad.

It was hard for me to have fun.^††^

^††^This item replaces “I thought that my life was bad” on the original recommended short form.

Anger:

I was so mad I did not want to talk to people.

I wanted to be alone because I was so angry.

I was so angry I felt like yelling at somebody.

I felt mad.

I had a bad temper.

I was so angry I felt like throwing something.

I felt upset.

I was angry when things didn't go my way.

Summed score to scale score translation for these short forms is in Table S1.

**Table S1: Summed Score to Scale Score Translation Table for the Recommended Short Forms**

|  | Anxiety | | Depressive Symptoms | | Anger | | |
| --- | --- | --- | --- | --- | --- | --- | --- |
| Summed Score | Scale Score | SD | Scale Score | SD | Scale Score | | SD |
| 0 | 33 | 6 | 35 | 6 | 30 | 6 | |
| 1 | 38 | 5 | 40 | 5 | 34 | 5 | |
| 2 | 41 | 5 | 43 | 4 | 37 | 4 | |
| 3 | 43 | 4 | 46 | 4 | 39 | 4 | |
| 4 | 45 | 4 | 47 | 4 | 42 | 4 | |
| 5 | 47 | 4 | 49 | 3 | 43 | 4 | |
| 6 | 48 | 4 | 51 | 3 | 45 | 4 | |
| 7 | 50 | 4 | 52 | 3 | 47 | 4 | |
| 8 | 51 | 4 | 53 | 3 | 48 | 4 | |
| 9 | 53 | 4 | 55 | 3 | 50 | 4 | |
| 10 | 54 | 4 | 56 | 3 | 51 | 4 | |
| 11 | 55 | 4 | 57 | 3 | 52 | 4 | |
| 12 | 56 | 4 | 58 | 3 | 54 | 4 | |
| 13 | 58 | 4 | 59 | 3 | 55 | 4 | |
| 14 | 59 | 4 | 60 | 3 | 57 | 4 | |
| 15 | 60 | 4 | 61 | 3 | 58 | 4 | |
| 16 | 61 | 4 | 62 | 3 | 59 | 4 | |
| 17 | 62 | 4 | 63 | 3 | 60 | 4 | |
| 18 | 63 | 4 | 64 | 3 | 62 | 4 | |
| 19 | 65 | 4 | 65 | 3 | 63 | 4 | |
| 20 | 66 | 4 | 66 | 3 | 64 | 4 | |
| 21 | 67 | 4 | 67 | 3 | 66 | 4 | |
| 22 | 68 | 4 | 68 | 3 | 67 | 4 | |
| 23 | 69 | 4 | 69 | 3 | 68 | 4 | |
| 24 | 71 | 4 | 70 | 3 | 70 | 4 | |
| 25 | 72 | 4 | 71 | 3 | 71 | 4 | |
| 26 | 73 | 4 | 73 | 3 | 72 | 4 | |
| 27 | 75 | 4 | 74 | 3 | 74 | 4 | |
| 28 | 76 | 4 | 75 | 3 | 75 | 4 | |
| 29 | 78 | 4 | 77 | 3 | 77 | 4 | |
| 30 | 79 | 4 | 78 | 4 | 79 | 4 | |
| 31 | 81 | 4 | 80 | 4 | 81 | 4 | |
| 32 | 84 | 5 | 83 | 4 | 84 | 4 | |

Scale scores are on a *T*-score scale; the values of SD are reported as conditional standard errors of measurement.
